# Antidiabetic Potential of Combined Cassiavera and Morel Berry Ethanol Extracts in Diabetic Rats

**DOI:** 10.1155/tswj/1526410

**Published:** 2025-05-20

**Authors:** Fauzan Azima, Wan Rosli Wan Ishak, Daimon Syukri, Rahmayani, Muhammad Iqbal, Yasmin Azzahra

**Affiliations:** ^1^Department of Food Technology and Agricultural Product, Universitas Andalas, Padang, Indonesia; ^2^Nutrition and Dietetics Programme, School of Health Sciences, Universiti Sains Malaysia, Kelantan, Malaysia

**Keywords:** antidiabetic, cassiavera (*Cinnamomum burmannii*), diabetes mellitus, morel berry (*Physalis angulata* L.)

## Abstract

This study investigated the effects of a combination of cassiavera and morel berry ethanol extracts (CMBEs) on diabetic rats, focusing on blood glucose levels, lipid profiles, immune and inflammatory markers, and pancreatic histopathology. Twenty male albino Wistar rats were divided into four groups: Group A (normal rats), Group B (diabetic rats), Group C (diabetic rats treated with metformin), and Group D (diabetic rats treated with CMBE). Parameters such as the IC_50_ of CMBE, body weight, blood glucose levels, lipid profile, immune markers (leukocyte percentage, total count, and macrophage activity), inflammatory markers (TNF-*α* and IL-6), and pancreatic histopathology of rats were assessed for 7 weeks. Statistical analysis was performed using one-way analysis of variance followed by Duncan's multiple range test, with a significance level of *α* = 5%. The results revealed an IC_50_ value of 18.5338 ppm for CMBE. Weight gain was observed in all groups except Group B, in which rats lost weight. CMBE was notably effective in lowering blood glucose levels in diabetic rats, with rats in Group D exhibiting higher HDL cholesterol levels and total leukocyte counts compared with those in Group C. Furthermore, CMBE significantly reduced TNF-*α* and IL-6 levels, exhibiting promising potential in promoting pancreatic repair. This study highlights the potential of a combination of Cassiavera and Morel Berry extract as an effective antidiabetic agent, suggesting it as a valuable addition to functional food formulations.

## 1. Introduction

Diabetes mellitus (DM) is one of the most frequent metabolic disorders, which is marked by elevated blood glucose. DM can be caused by a lack of insulin in the circulatory system, either due to a lack of insulin production or because the body is unresponsive to the existing insulin [1]. Type 1 DM is an autoimmune condition in which the immune system damages the cells in the pancreas that produce insulin, making it difficult for the body to properly regulate blood sugar levels. Type 2 diabetes mellitus (T2DM), which is more common, occurs when the body does not produce enough insulin or the body's cells become insensitive to insulin. This condition is usually influenced by factors such as obesity, poor diet, and lack of physical activity. Apart from that, various environmental and lifestyle factors can also influence the onset of diabetes. There is also a correlation with genetic predisposition, insulin resistance, insulin deficiency, and hyperglycemia in DM. The prevalence of DM continues to increase worldwide and represents a significant health burden [2].

Several types of oral antidiabetic drugs can be used to treat DM, for example, sulfonylureas, metformin, glibenclamide, thiazolidinediones, and also a combination of drugs [3]. However, using these drugs for an extended period can cause severe side effects, including hypoglycemia, digestive disorders, abdominal swelling, liver damage, and complications of cardiovascular problems [4]. Therefore, the consumption of antidiabetic drugs needs to be reduced and replaced with natural products.

Cassiavera (*Cinnamomum burmannii*) is an Indonesian cinnamon that is thought to be able to treat DM, especially T2DM. Cassiavera contains active compounds, such as cinnamaldehyde, cinnamic acid, polyphenols, and flavonoids, which have hypoglycaemic effects and increase insulin sensitivity [5]. Moreover, cassiavera is believed to influence several insulin signaling pathways, including the insulin receptor, peroxisome proliferator–activated receptor (PPAR), glucose transporter 4, *α*-glucosidase activity, and glucose transporter-1. The significant compounds that affect hypoglycaemic activity are methyl hydroxy chalcone polymer (MHCP), cinnamaldehyde, and A-type proanthocyanidin polymer [6].

Morel berry (*Physalis angulata* L.) contains polyphenols and flavonoids that can act as antioxidants. The antioxidant properties of flavonoids can strengthen regeneration by inhibiting the effects of free radicals. Other active components such as physalins, withanolides, phytosterols, and unsaturated fatty acids such as oleic acid and linoleic acid in morel berry also provide antioxidant effects and reduce blood glucose levels. A 150-mg/kg BW morel berry ethanol extract can lower blood glucose levels in albino rats with the best dose. The administration of this extract is also known to have the ability to repair alloxan-induced pancreatic *β*-cell damage in diabetic rats [7]. Physalin, a steroid lactone found in morel berry, can inhibit the activation of NF-*κ*B. NF-*κ*B is a protein involved in inflammatory processes and immune responses, which is often implicated in the development of diabetes. In the context of diabetes, NF-*κ*B activation can worsen glucose imbalance through inflammation and insulin resistance. Therefore, physalin's ability to inhibit NF-*κ*B suggests that it may help maintain glucose homeostasis in the body, which is crucial for managing diabetes [8].

Functional foods provide essential nutrients and additional health benefits, such as enhancing immune function, preventing diseases, or supporting heart health. They contain bioactive compounds like fiber, vitamins, probiotics, and antioxidants, offering health effects beyond essential nutrition [9] Preclinical testing on the potential of the ingredients is necessary in developing functional foods. Testing on the animal model needs to be carried out to observe the biological response to the effects of product consumption. There is no research article on the effect of mixed cassiavera and morel berry in diabetic rats. This research was intended to observe the ability of a mixture of cassiavera and morel berry as an antidiabetic agent in alloxan-induced rats.

## 2. Materials and Methods

### 2.1. Preparation of Mixed Cassiavera–Morel Berry Extract (CPE)

Cassiavera AA grade (Sumatera Tropical Spices, Indonesia) was ground using a grinder (Wilman, Indonesia). Dried morel berry (6% db) was ground with a blender (Philip, Holland) for 3 min. Based on authentication verification conducted at the Herbarium of Universitas Andalas, cassiavera belongs to the Lauraceae family with the species *Cinnamomum burmannii* (Nees and T. Nees) Blume, while morel berry belongs to the Solanaceae family with the species *Physalis angulata* L. Both were sieved through 40-mesh sieves. Each powder was macerated using 96% ethanol (Novalindo, Indonesia) with a powder-to-solvent ratio of 1:5 (24 h, occasional stirring). The maceration was filtered and concentrated with a rotary evaporator (Buchi, Switzerland) at 40°C. The extracts were mixed with a ratio of cassiavera and morel berry of 7:3. Then, CMBE was stored in a dark bottle for further analysis [10].

### 2.2. Antioxidant Activity (IC_50_)

The procedure was adapted and modified according to several references [11–15]. About 0.1 g of sample was put into a 100-mL measuring flask, and methanol was added to the mark. Then, 2 mL of the sample at each dilution was taken, and 1 mL of 2,2-diphenyl-1-picrylhydrazyl (DPPH) (the concentration used was 200 *μ*M) was added (minimal light and a closed room). After 15 min, it was read using a UV-vis spectrophotometer (Shimadzu 1800, Japan) at 517 nm. The reading results were then entered into the % inhibition formula, and then a curve was created to get a linear regression.

### 2.3. Animal and Experimental Design

Male albino Wistar rats (150–200 g, 2–3 months) were obtained from Animal House Andalas University, Indonesia—the division of groups adhered to guidelines for metabolic tolerance tests in mice [16]. The rats were acclimatized and fed commercial (CitraFeed, RatBio) chow and water ad libitum (25°C ± 2°C, 12-hour light/dark rotation). Following a 7-day adaptation period, the rats received a single intraperitoneal injection of 100 mg/kg BW of alloxan monohydrate 98% A7413-10G (Aldrich, 102596749). After administering 36 injections to experimental rats, blood glucose levels were checked using the postprandial glucose method through the rat's tail. Blood sampling was done through blood capillaries in the tail of rats. Rats with postprandial blood glucose levels greater than 200 mg/dL were selected and randomly divided into four groups. The treatment groups for the rats can be seen in Table 1. Treatments were administered every 2 days, with dosage adjusted according to body weight, via oral administration. The experiment lasted for 7 weeks.

The dose of drugs given is the result of the conversion of human doses to rats. The determination of the extract dose is also determined based on the IC_50_ value of the drug compared to the IC_50_ of the extract. The basic principle of determining the dose is to ensure that the plant extract can produce an effect that is equivalent to or even greater than the standard drug.

### 2.4. Postprandial Glucose Test and Blood Serum Sampling

Rats were fasted for 2 h and anesthetized with diethyl ether (Merck). Blood was collected through the orbital sinus of the rat's eye using microhematocrit tubes (Nris, Denmark). Blood was placed on the glucose strip to check the postprandial glucose. Blood was also collected in a vacuum blood collection tube gel separator filled with ±2 mL of blood. The collected blood was gently mixed and centrifuged at 4000 rpm for 10 min (Hettich-Centrifuge Benchtop EBA 20, Germany). After centrifugation, the blood serum will be located at the top of the gel separator. The blood serum was put into a microtube (Onemed, Indonesia) and stored at −80°C until used for analysis [17]. The blood serum was used for lipid profile and inflammatory indicators.

### 2.5. Lipid Profile

The samples used were blood serum at the end of 7 weeks. Total cholesterol (TC), triglyceride (TG), and high-density lipoprotein (HDL) were analyzed by the CHOD-PAP method (enzymatic photometric test). Low-density lipoprotein (LDL) was calculated by the Friedewald formula. Ten microliters of distilled water, standard, and serum samples was added to different tubes. A 1000 *μ*L of cholesterol reagent (Diasys) was added to each tube, then incubated (10 min, 37°C ± 0.5°C). Then, value levels are determined using a microlab 300 (vital scientific, Germany) at a wavelength of 546 nm.

### 2.6. Immunological Indicators

Observations were conducted on the percentage of leukocyte cells (neutrophils stem, neutrophil segments, monocytes, lymphocytes, and eosinophil cells) extracted from the tails of mice. The overall count of leukocyte cells, as well as the activity and capacity of macrophage cells, was assessed according to the specified protocol [18].

### 2.7. Inflammatory Indicators

Inflammatory markers were measured using BT LAB's instructions. A 50-*μ*L standard solution was added to the standard wells, while 40 *μ*L of the sample and 10 *μ*L of anti-IL-6 or anti-TNF-*α* antibody were added to the sample wells. Streptavidin HRP (50 *μ*L) was added to the sample and standard wells and then mixed and incubated (Biosan PST-100HL, Germany) for 60 min at 37°C. After washing the plate five times with wash buffer (2-morpholinoethanesulfonic acid and monohydrate buffer), 50 *μ*L of Substrate A and B was added, and the plate was incubated for 10 min at 37°C. A stop solution (50 *μ*L) was added, and the optical density was measured at 450 nm by a microplate reader (xMark BIO-RAD, California).

### 2.8. Histopathology of the Pancreas

The procedure was adapted from El-esawy et al. [19] with modification. The surgical area was prepared by placing the rat in a supine position (lying on its back) on a surgical board, with its limbs secured using pins to facilitate access to the abdominal cavity. The incision area was then sterilized with 70% ethanol or povidone–iodine to prevent contamination. The initial incision was made by cutting the abdominal skin longitudinally using surgical scissors or a scalpel until the muscle layer was exposed. The abdominal cavity was carefully opened by separating the muscles to avoid damage to internal organs. The pancreas was identified based on its location near the stomach, spleen, and duodenum. The pancreatic tissue was then carefully excised using fine forceps and surgical scissors, ensuring minimal damage to its histological structure. The excised pancreas was immediately rinsed with a physiological solution (PBS or saline) to remove any remaining blood and debris. Finally, the tissue was fixed in a 10% buffered formalin solution to preserve cellular structures and prevent tissue degradation. Paraffin sections of pancreatic tissue were stained with hematoxylin–eosin after removing paraffin. Each islet was examined and scored. The area of islets of Langerhans was measured in five fields of view at 10x magnification of the objective lens, reported as mean islet area in square micrometers with a CX 33 microscope (Olympus, Japan), a 3.1 MP CMOS Sony Beta camera, and the beta view program. Cell damage was scored semiquantitatively in four levels: Score 0 (normal histology, no sign of damage, or cell abnormality, < 25%), Score 2 (mild damage, 25%–50%), Score 3 (moderate damage, 50%–75%), and Score 4 (severe damage, > 75%). The final score of each sample was obtained by averaging the scores observed in each islet.

### 2.9. Statistical Analysis

Statistical comparisons were performed using the IBM SPSS Statistics 22 program, employing one-way analysis of variance (ANOVA) and DMRT with *α* = 5%.

## 3. Results and Discussion

### 3.1. Antioxidant Activity (IC_50_)

**Figure 1:**
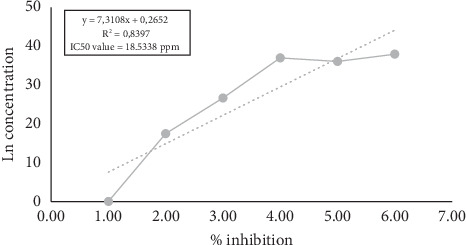
Graph of IC_50_ of mixed cassiavera–morel berry.

The IC_50_ value is a concentration that can inhibit the activity of DPPH by 50%. DPPH is an organic compound in the form of a dark crystalline powder and is a stable free radical at room temperature. The research found that the average IC_50_ value of the mixed cassiavera and morel berry extract was 18.5338 ppm (Figure 1). Previous studies have reported that cassiavera extract had an antioxidant activity of 88.50%, morel berry extract had 39.69%, and the mixed extract (7:3 ratio) had 89.34%, indicating a potential synergistic effect [20]. It means that the antioxidant activity of CMBE was classified as very strong. Based on the results of research conducted by Wellyalina et al. [21], cassiavera powder contained active components in the form of phenolics, flavonoids, terpenoids, and saponins. The results of GC-MS chromatogram analysis showed that cinnamaldehyde was the highest element in the active fraction (4.52% in cinnamon powder). The flavonoids in cassiavera consist of procyanidins and phenolic components and have other specific antioxidants such as epicatechin, camphene, eugenol, y-terpinene, phenol, and tannins [22]. These components help combat oxidants, increase potential insulin activity, and enhance glucose and lipid metabolism. On the other hand, morel berries contain alkaloids, flavonoids, saponins, polyphenols, steroids, triterpenoids, monoterpenoids, and sesquiterpenoids. Both flavonoid and polyphenol compounds are claimed to be very potent antioxidants [7].

### 3.2. Body Weight

**Figure 2:**
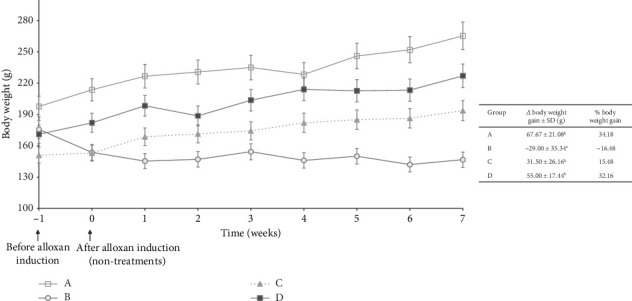
Graph of body weight (0 week is a week after alloxan induction and without treatment for any groups).

Figure 2 shows the average weight change in rats for each group, starting from Week −1 (initial weight before alloxan induction) to Week 0 (after alloxan induction without treatment) and continuing over 7 weeks of treatment. The rat's body weight was observed once a week. Groups A, C, and D showed an increase in body weight, while the group with diabetes induction (B) experienced a decrease in body weight.

The most significant percent weight gain in the sequence was Group A (34.18%), and the smallest was Group B (−16.48%). The negative value indicated that Group B experienced weight loss. The change in body weight of rats in Group B was statistically significantly different from the other groups. Weight loss is caused by insulin deficiency or insulin resistance, which affects fat and protein metabolism. As a result, glucose cannot move normally through the blood into the body's cells, which interferes with energy supply. The body tends to break down fat and muscle to obtain energy, which can lead to unwanted weight loss [23].

The administration of cassiavera extract to diabetic rats has a significant effect in maintaining the stability of diabetic body weight so as not to experience drastic weight loss. This data was in line with other research that stated that the administration of cassiavera extract could maintain the body weight of diabetic rats and improve lipid metabolism in diabetic rats. The beneficial effects of cassiavera extract on body weight in diabetic rats may be due to several mechanisms. One of which is the ability of cassiavera to increase glucose utilization in the body and decrease glucose absorption by the intestine. Cassiavera is also known to have anti-inflammatory and antioxidant properties. It may protect the cells of the pancreas that produce insulin [24].

### 3.3. Blood Glucose

After administration of alloxan (Week 0), the rats experienced an increase in blood glucose to above normal limits (postprandial glucose > 200 mg/dL). This data indicated that alloxan worked well to cause diabetes in the rats (other than Group A). After Day 0, rats were treated in Groups C and D orally every 2 days. In Week 1, Groups C and D experienced decreased blood glucose levels. However, the blood glucose of Group C was still above the standard limit (> 200 mg/dL). When blood glucose was checked from 1 week until 7 weeks, both were stable, below the standard limit.

**Figure 3:**
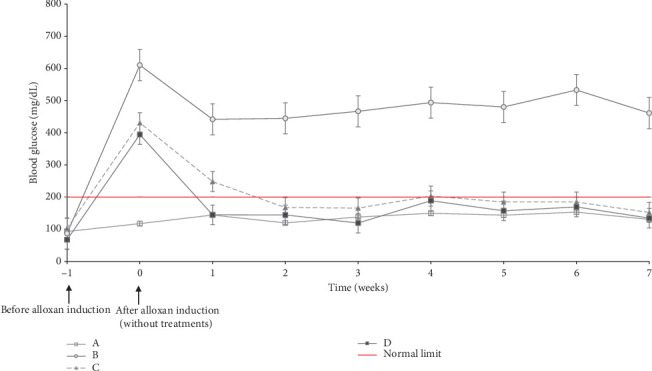
Graph of blood glucose level (0 week is a week after alloxan induction and without treatment for any groups).

Figure 3 shows a reduction in blood glucose levels in Groups C and D. Diabetic rats treated with metformin (Group C) successfully lowered their blood glucose levels to the normal range within 2 weeks. Meanwhile, rats treated with CMBE (Group D) could quickly bring their blood glucose levels to the normal range within just 1 week. Alloxan induction in rats caused damage to pancreatic beta cells, so insulin production is disrupted and decreased. Low insulin availability means glucose cannot enter cells for energy use [25]. This condition is caused by low insulin levels or insulin resistance. This action prevents the rat's body from breaking down glucose into energy, causing hunger (polyphagia). Polyphagia does not disappear even after consuming food. As a result, rats will eat more significantly than usual, which will cause an increase in blood glucose.

Mixed cassiavera extract and morel berry effectively reduced blood glucose levels. Cassiavera has antidiabetic potential with active compounds such as MHCPs, cinnamaldehyde, cinnamic acid, polyphenols, and flavonoids [26], which significantly regulate insulin signaling and pancreatic function [26]. Cinnamaldehyde metabolites from cinnamon are essential in managing insulin release and regulating the activity of insulin-related enzymes, such as protein–tyrosine phosphatase 1B and insulin receptor kinase [27].

Cinnamaldehyde works by increasing the sensitivity of muscle tissue and adipose tissue to insulin, improving glucose metabolism, reducing hepatic glucose production, and increasing islet function in the pancreas [28, 29]. MHCP works by increasing glucose metabolism and tissue sensitivity to insulin [25]. Cinnamaldehyde and cinnamic acid enhance insulin sensitivity through distinct mechanisms involving various molecular pathways. Cinnamaldehyde activates PPARs *δ* and *γ*, which play crucial roles in glucose metabolism and lipid regulation. This activation leads to increased expression of target genes associated with insulin sensitivity and fatty acid oxidation in adipose tissue and skeletal muscle [30]. Additionally, cinnamaldehyde primes Akt2, a key protein in the insulin signaling pathway, enhancing its phosphorylation and activity, which is vital for an effective insulin response [31, 32]. Cinnamic acid, particularly in its phospholipid derivatives, has been shown to restore insulin sensitivity in adipocytes by improving insulin-stimulated glucose uptake, especially in insulin-resistant conditions. Morel berry exhibits anti-inflammatory activity by blocking the cyclooxygenase pathway, thereby reducing prostaglandin E2. In addition, morel berry also blocks the lipo-oxygenase pathway, reducing leukotriene B4, which is a chemotactic agent [33].

### 3.4. Lipid Profile

**Figure 4:**
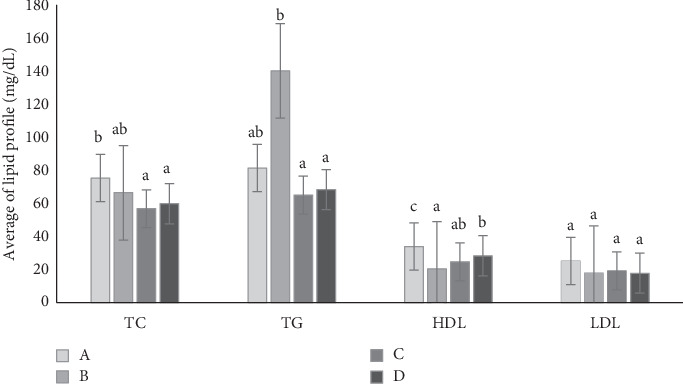
Histogram of lipid profile in rat's blood serum. Different letters at the top of the bar mean that there is a significantly difference at =5%.

Figure 4 shows the lipid profile of the rats at the end of the observation period (end of Week 7). Diabetes increases TG levels while decreasing HDL levels in rats. The administration of metformin and the mixed cassiavera and ciplukan extract to diabetic rats showed similar effects in reducing TG and TC and increasing HDL levels, which were similar to the levels seen in normal rats. CMBE demonstrates potential comparable to metformin, particularly in lowering TGs and TC, and is even more effective in raising HDL levels than metformin. Several studies show that metformin can reduce lipid synthesis in the liver, TG, and TC levels in diabetic rats. In addition, metformin can also increase fatty acid oxidation in adipose tissue and skeletal muscle, thereby reducing fat accumulation and increasing the use of fatty acids as an energy source. Another mechanism was activating AMPK (adenosine monophosphate protein kinase), which can improve insulin sensitivity and reduce glucose production in the liver [34].

The flavonoids and polyphenols in morel berry extract have antioxidant activity, which can strengthen regeneration, inhibit free radicals, and reduce cholesterol. Hyperglycemia is associated with dysfunction of fat metabolism. Insulin has the most critical influence on lipid metabolism, reducing lipolysis in adipose tissue so that fatty acid levels increase. Insulin also influences triacylglycerol synthesis and stimulates the absorption of TGs into muscle and adipose tissue in the blood. Insulin deficiency affects fat metabolism, which can be a risk factor in vascular complications associated with DM [35].

Administering morel berry extract combined with cassiavera extract can reduce total glycerol in the blood and increase HDL in treated diabetic rats compared to Group B (diabetic rat nontreatment). Novitasari et al. [29] published a review article about morel berry as a medical plant. Based on the review article, administration of 400 mg/kg of morel berry aqueous extract can repair lipid profile, reduce TG values, and increase HDL value after 8 days of consumption. The LDL value also decreased, but it was not significantly different from that of diabetic rats. Besides that, the administration of cassiavera water extract can provide hypolipidemic effects in the form of a decrease in TC, TG, and LDL and an increase in HDL in the plasma of rats induced by alloxan.

### 3.5. TNF-*α* and IL-6 Levels

Based on the graph of changes in TNF-*α* during treatment, it can be seen that Group A, Group C, and Group D experienced ups and downs over 4 weeks (Table 2). The negative values indicate a decreasing TNF-*α* value. Group B had an increased value of TNF-*α* with a percentage increase of 89.3%.  Table 3 indicates that the IL-6 value was similar to the TNF-*α* value. In Group B, the rats experienced inflammation, reaching 45.45%, while Groups A and D showed a decrease. The study showed that the administration of alloxan in rats may significantly impact the increase in inflammation, particularly TNF-*α* and IL-6.

Cinnamon inhibits TNF-*α*'s release from neutrophils and reduces the expression of proinflammatory cytokine genes, thereby reducing the production of proinflammatory cytokines, especially TNF-*α*. The cinnamaldehyde content in cassiavera can also reduce serum nitric oxide (NO), TNF-*α*, and PGE2 levels, thus showing its anti-inflammatory properties. The flavonoid content in cassiavera stops excessive production of lipoproteins containing ApoB-48 (apolipoprotein B-48 and chylomicron lipoproteins) and TG levels in postprandial serum. Increased amounts of ApoB-48 can trigger the release of proinflammatory substances, which may contribute to the inflammatory response. Additionally, lipoproteins with ApoB-48 can interact with immune cells and immune system components, triggering an inflammatory [36, 37].

The amount of TNF-*α* is a protein signal molecule in the form of a proinflammatory cytokine that plays a crucial role in regulating the body's inflammatory response. Cassiavera contains polyphenols that can increase mRNA levels and tristetraprolin (TTP) protein gene expression. An increase in TTP contributes to deciphering TNF-*α* mRNA so that the number of TNF-*α* decreases [38].

Morel berry ethanol extract reduced the production of nitrite, IL-6, IL-12, and TNF-*α* by activated macrophages without damaging activated cells. Steroid compounds such as physalins B, F, and G isolated from morel berry can reduce NO and interferon *γ* levels. Both physalins B and F isolated from the stems of morel berry showed anti-inflammatory effects by activating the receptors of glucocorticoids. Flavonoid compounds have also been found on leaves and other plant parts [35]. Morel berry ethanol extract can reduce the production of nitrite, IL-6, IL-12, and TNF-*α* by activated macrophages without damaging activated cells [39]. A study showed that physalin D can reduce cytokine release and protein accumulation associated with hyperglycemia, suppressing chronic inflammatory responses. Physalin D may protect the body's tissues from damage by suppressing inflammation, often in organs prone to DM complications, such as the kidneys and nerves [29].

### 3.6. Percentage of Leukocytes

Leukocyte percentage in the diabetic group was mainly found in the neutrophil and lymphocyte types (Table 4). The highest percentage of leukocytes was neutrophils, which reached 23% in Group B. It may be due to the involvement of these cells in the process of phagocytosis against different antigens.

**Figure 5:**
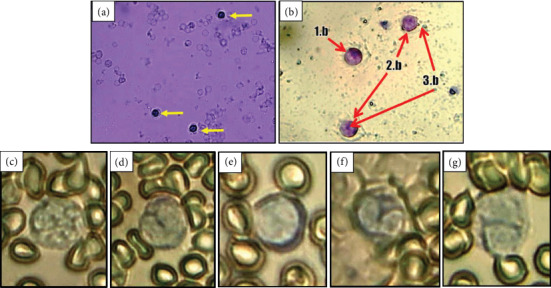
(a) Leukocyte cells (yellow circle) of rat at 400x after staining with Turk stain, (b) macrophage cells of rat (1.b = macrophage, 2.b = active macrophage, and 3.b = *S. aureus*), (c) segment neutrophils, (d) stem neutrophil, (e) lymphocyte, (f) monocytes, and (g) eosinophils.

Group D had the highest percentage of lymphocytes. Lymphocytes help to alert the body to the presence of antigens or foreign substances. Increased neutrophils may also result from decreased phagocytic activity in hyperglycemia and increased hematopoietic activity after the release of neutrophil granules by exocytosis to lyse antigens extracellularly [14]. Meanwhile, the percentage of lymphocytes was the highest in the group of diabetic rats that were given CMBE. The meager percentage of monocytes and eosinophils in rats is because monocytes have differentiated into macrophages, while eosinophils play a role if allergies occur. The exact shape of each leukocyte cell can be seen in Figure 5.

### 3.7. Number of Total Leukocytes (Leukocyte Total)

**Figure 6:**
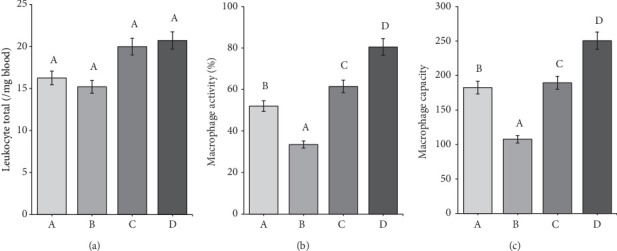
Histogram of (a) leukocyte total, (b) macrophage activity, and (c) macrophage capacity in rat's blood. Different letters at the top of the bar mean that there is a significantly difference at =5%.

Leukocytes in rats ranged from 15,250 to 20,700/*μ*L blood (Figure 6a). Group B had the lowest leukocyte total among all groups. The low leukocyte total in Group B was thought to be due to the diabetic rats' experience of a decline in their immune system after 7 weeks.

According to Rehman et al. [40], decreasing immunity is caused in response to the administration of natural alkylating antineoplastic agents (streptozotocin and alloxan), resulting in the formation of T2DM. Decreasing the immune system can also affect the number and quality of leukocyte cells and weaken the body's ability to protect itself from infection.

Meanwhile, the leukocyte total in the D group amounted to 20,700/*μ*L of blood. The high leukocyte total may be related to secondary metabolite compounds in the CMBE. Giving morel berry leaf extract to tilapia by injection positively increased the total numbers of leukocytes. Morel berries contain saponin and flavonoid compounds with immunostimulatory properties that evoke an immune response [29].

### 3.8. Phagocytic Activity of Macrophages

Macrophages, found in various tissues, some body cavities, and around some mucosal surfaces, are an essential part of the innate immune system for the body's defense against many pathogens and cancer [41]. Macrophages have three critical functions: immunomodulating, phagocytosing, and antigen-presenting. In several pathophysiological conditions, they are essential for a normal immune response [42].

The average macrophage activity in the rat ranged from 34% to 80% (Figure 6b). The lowest macrophage activity was in Group B, namely, 34%. It can be due to a decline in the rat's immune system for 7 weeks, which resulted in macrophages being unable to phagocytose optimally and finding it challenging to provide defense when infection occurs. Diabetic rats experience inflammation and immune deficiency due to reduced macrophage phagocytic activity, making them susceptible to infection. Decreased macrophage activity may also be caused by hyperglycemia in DM, leading to oxidative stress and glycation, which causes cell death [18]. Meanwhile, Group A and Group C had macrophage activity that was not much different, namely, 53%–62%.

The highest macrophage activity was in Group D, up to 80%. CMBE can potentially increase macrophage activity and improve the immune system in diabetic rats. The high activity of macrophages was due to the immunomodulatory properties of morel berry and cassiavera. Some physalins in morel berries, such as B and F, strongly affect activated macrophages [39]. Meanwhile, cinnamon's water extract affects monocyte differentiation into macrophages and scavenger receptor activity. The extract was found to increase the phagocytic activity of macrophages [43].

### 3.9. Macrophage Capacity

The average macrophage capacity in the rat ranged from 105 to 254 cells. The ANOVA results showed that all of the groups had significant differences from each other. Group B had the lowest capacity of macrophages. Macrophage capacity refers to the ability of macrophages to carry out their functions, such as phagocytosis. The greater the macrophage capacity, the faster the macrophages carry out phagocytosis.  Figure 6c shows that Group D (CMBE-treated rat) had a higher macrophage capacity than the other groups. This result was thought to be due to secondary metabolites, which may act as immunomodulators in morel berry and cassava.

Meanwhile, the macrophage capacity in the diabetic rat group was only 105 cells, the smallest among all groups. The low capacity of macrophages in the diabetic rat group was due to a decrease in the immune system in diabetic rats. According to Leniseptaria et al. [44], DM sufferers will experience defects in immune cells, decreasing macrophage's phagocytic capacity.

### 3.10. Histopathology of the Pancreas

The histopathology of the pancreas of diabetic rats was taken after 7 weeks of treatment. Observations were made at 10x and 40x magnification. At 10x magnification of the objective lens, the shape and area of the islets of Langerhans were observed. At 40x magnification of the objective lens, observations were made regarding damage to the histopathology of Langerhans islet endocrine cells.

**Figure 7:**
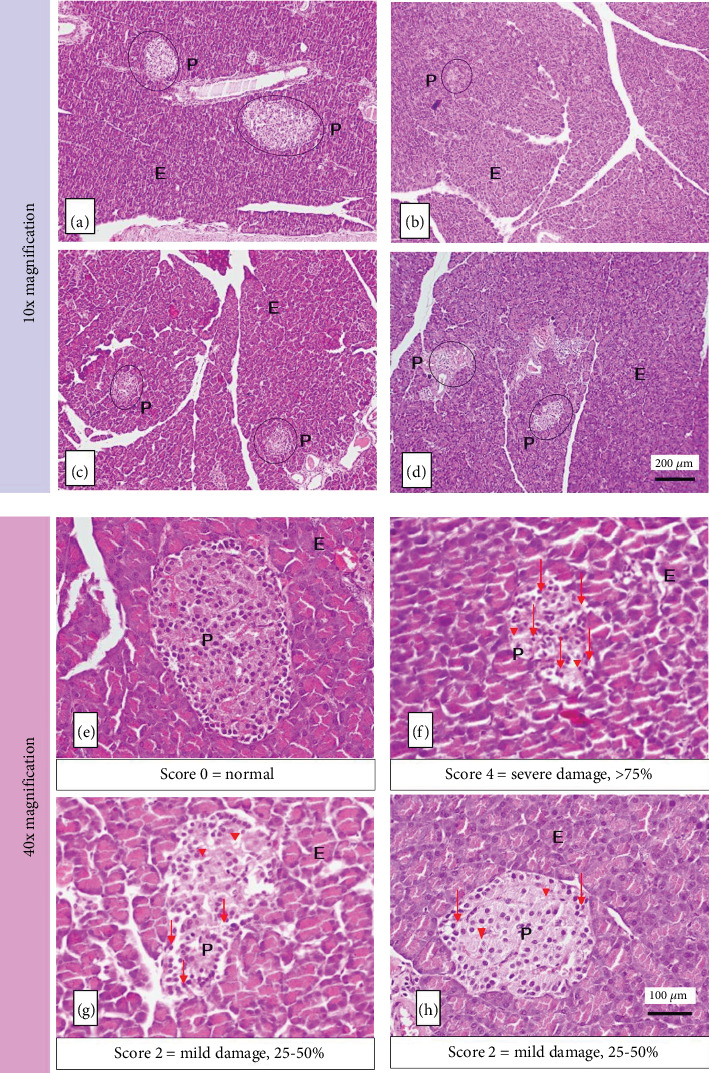
Histology of the pancreas of rats showing the exocrine component (E) and endocrine component of Langerhans islets (P). (a, e) Normal rat, (b, f) diabetic rat, (c, g) metformin-treated rat, and (d, h) CMBE-treated rat. Group B shows islets of Langerhans atrophy. Endocrine cells showed signs of degeneration and necrosis, characterized by cells with shrinking pyknotic nuclei (red downward arrow) and lysis of cells (red down-pointing triangle). Metformin-treated rats and CMBE-treated rats showed a decreased proportion of islets of Langerhans damage.

Figure 7 shows that Group A had a regular shape and was classified in the normal category with a damage score of 0, the average area of Langerhans islets of 211577.31 ± 7357.47 mm^2^. The group of alloxan-induced rats (B, C, and D) experienced damage and reduction in the size of the islets of Langerhans, with the highest damage score in Group B (Score 4, signs of damage > 75%). At the same time, Group C and Group D were not significantly different (Score 2, signs of damage 25%–50%).  Table 5 shows that the results of these groups were better than those of Group B. This result indicates that the islets of Langerhans have been repaired with a moderate damage score.

Alloxan-induced rats showed atrophy (shrinkage) of the islets of Langerhans. It experienced abnormal changes in cell structure (degeneration) and cell death (necrosis), which is characterized by cell nuclei undergoing pycnosis so that the color of the cell nucleus becomes darker, the boundaries between cells are not clear, the cytoplasm is pale, and cells are lysed. Alloxan produces oxidative stress that causes damage to pancreatic cell membranes through increased permeability, resulting in decreased insulin production [45].

MHCP content in cassiavera has an insulin-like activity that can stimulate glucose oxidation. Cassiavera extract can also inhibit glycogen synthase kinase-3 and dephosphorylate insulin receptors, increasing insulin sensitivity [6, 22, 46]. Meanwhile, morel berry contains terpenoid compounds that can stimulate cell regeneration in the islets of Langerhans. This process causes cell damage to the islets of Langerhans (especially *β*-cells), which can be gradually reduced, and the number returns to normal. Ethanol extract of morel berry leaves has antidiabetic activity in the dose range between 10 and 100 mg/kg BW [29, 47].

## 4. Conclusions

This research showed that the combination of cassiavera extract and morel berry was effective in reducing blood glucose levels, reducing TNF-*α* and IL-6 levels, increasing HDL, recovering the body's immunity, recovering and reducing damage in the Langerhans islet of diabetic rats, maintaining and increasing the body weight of rats, and maintaining the glucose within normal limits. The administration of 90 mg/kg BW of mixed cassiavera and morel berry (7:3) to diabetic male albino Wistar rats can act as an antidiabetic and has the future potential to be further processed as a dietary functional food for diabetes therapy to replace the use of drugs.

## Figures and Tables

**Table 1:** Group treatment.

**Group**	**Description**
A	Control negative, normal rats without treatment
B	Control positive, diabetic rats without treatment
C	Diabetic rats with metformin treated orally at a dosage of 45 mg/kg BW
D	Diabetic rats with CMBE treated (cassiavera–morel berry extract) orally at a dosage of 90 mg/kg BW

**Table 2:** Average of TNF-*α*, delta, and percentage increasing of TNF-*α* of rat's blood serum.

**Group**	**A** **v** **e** **r** **a** **g** **e** ± **SD (pg/mL)**					**Change value of TNF-*α***	
	**0**	**1**	**2**	**3**	**4**	Δ	**%**
A	279.42 ± 14.12	262.11 ± 24.50	283.23 ± 5.63	249.15 ± 14.36	264.36 ± 8.17	−15.06^a^	−5.39
B	153.11 ± 10.89	200.43 ± 20.84	255.35 ± 18.31	263.95 ± 17.32	289.57 ± 28.72	136.46^b^	89.13
C	246.62 ± 12.64	199.49 ± 22.58	215.26 ± 25.75	206.15 ± 21.12	220.89 ± 29.12	−25.72^a^	−10.43
D	213.95 ± 16.03	200.43 ± 26.00	188.60 ± 25.43	204.37 ± 38.25	192.17 ± 32.22	−21.78^a^	−10.18

*Note:* Columns with different unequal letters differ significantly at *α* = 5% by DMRT (0 week means a week after alloxan induction without treatment in any groups).

**Table 3:** Average of IL-6, delta, and percentage increasing of IL-6 of rat's blood serum.

**Group**	**A** **v** **e** **r** **a** **g** **e** of **IL**‐6 ± **SD (pg/mL)**				**Change value of IL-6**	
	**0 week**	**2 weeks**	**4 weeks**	**6 weeks**	Δ	**%**
A	77.971 ± 7.88	79.495 ± 5.01	67.630 ± 1.35	69.143 ± 7.02	−8.828 ± 4.73	−11.32^a^
B	69.899 ± 8.10	88.050 ± 2.70	84.273 ± 2.72	101.671 ± 8.99	31.772 ± 4.72	45.45^b^
D	68.386 ± 4.90	71.916 ± 4.56	59.812 ± 3.81	57.291 ± 8.96	−11.095 ± 6.65	−16.22^a^

*Note:* Columns with different unequal letters differ significantly at *α* = 5% by DMRT (0 week means a week after alloxan induction without treatment in any groups).

**Table 4:** Average of percentage of leukocytes in rat's blood.

**Group**	**A** **v** **e** **r** **a** **g** **e** **percentage** of **leukocyte** **cells** ± **SD (%)**				
	**Segment neutrophils**	**Stem neutrophils**	**Lymphocytes**	**Monocytes**	**Eosinophils**
A	49 ± 2.83^b^	23 ± 2.12^b^	18 ± 2.12^b^	4 ± 1.41	7 ± 2.83
B	82 ± 2.83^d^	7 ± 1.41^a^	5 ± 1.41^a^	3 ± 1.41	5 ± 0.71
C	66.5 ± 1.71^c^	8 ± 1.00^a^	17 ± 0.80^b^	4 ± 2.12	6 ± 0.71
D	35 ± 1.41^a^	6 ± 2.12^a^	55 ± 1.41^c^	4 ± 0.71	4 ± 1.41

*Note:* Columns with different unequal letters differ significantly at *α* = 5% by DMRT.

**Table 5:** Histopathology of rat pancreas (average of area and damage score of Langerhans islets).

**Group**	**Average of area of Langerhans islets (*μ*m** ^**2**^ **)**	**Average damage score of Langerhans islets**
A	21157.31 ± 7357.47^b^	0.00 ± 0.00^a^
B	3812.91 ± 747.23^a^	3.33 ± 0.23^c^
C	12129.74 ± 1771.92^a^	2.07 ± 0.23^b^
D	10608.6 ± 4613.9^a^	2.00 ± 0.20^b^

*Note:* Columns with different unequal letters differ significantly at *α* = 5% by DMRT.

## Data Availability

The data that support the findings of this study are available from the corresponding author upon reasonable request.
